# PP242 Counteracts Glioblastoma Cell Proliferation, Migration, Invasiveness and Stemness Properties by Inhibiting mTORC2/AKT

**DOI:** 10.3389/fncel.2018.00099

**Published:** 2018-04-10

**Authors:** Carmen Mecca, Ileana Giambanco, Stefano Bruscoli, Oxana Bereshchenko, Bernard Fioretti, Carlo Riccardi, Rosario Donato, Cataldo Arcuri

**Affiliations:** ^1^Department of Experimental Medicine, Perugia Medical School, University of Perugia, Perugia, Italy; ^2^Department of Medicine, Perugia Medical School, University of Perugia, Perugia, Italy; ^3^Department of Chemistry, Biology and Biotechnology, University of Perugia, Perugia, Italy; ^4^Centro Universitario per la Ricerca sulla Genomica Funzionale, University of Perugia, Perugia, Italy

**Keywords:** glioblastoma, mTOR, mTORC2, rapamycin, PP242

## Abstract

Glioblastoma multiforme (GBM) is the most malignant brain tumor and is associated with poor prognosis due to its thorny localization, lack of efficacious therapies and complex biology. Among the numerous pathways driving GBM biology studied so far, PTEN/phosphatidylinositol-4,5-bisphosphate 3-kinase (PI3K)/AKT/mechanistic target of rapamycin (mTOR) signaling plays a pivotal role, as it controls cell survival, proliferation and metabolism and is involved in stem cell maintenance. In front of recent and numerous evidences highlighting mTOR upregulation in GBM, all the strategies developed to inhibit this pathway have been substantially unsuccessful. Our study focused on mTOR complex 2 (mTORC2) to understand its involvement in GBM cell growth, proliferation, migration and invasiveness. We utilized an *in vitro* model, characterized by various genetic alterations (i.e., GL15, U257, U87MG and U118MG cell lines) in order to achieve the clonal heterogeneity observed *in vivo*. Additionally, being the U87MG cell line endowed with glioblastoma stem cells (GSCs), we also investigated the role of the PTEN/PI3K/AKT/mTOR pathway in this specific cell population, which is responsible for GBM relapse. We provide further insights that explain the reasons for the failure of numerous clinical trials conducted to date targeting PI3K or mTOR complex 1 (mTORC1) with rapamycin and its analogs. Additionally, we show that mTORC2 might represent a potential clinically valuable target for GBM treatment, as proliferation, migration and GSC maintenance appear to be mTORC2-dependent. In this context, we demonstrate that the novel ATP-competitive mTOR inhibitor PP242 effectively targets both mTORC1 and mTORC2 activation and counteracts cell proliferation via the induction of high autophagy levels, besides reducing cell migration, invasiveness and stemness properties.

## Introduction

Glioblastoma multiformes (GBMs) are the most malignant and more frequent gliomas (about 54% of all astrocytic tumors). The median age of patients at diagnosis is 64 years and the male:female ratio is 1.58:1. GBMs are composed of spindle or rounded cells and typical multinucleated giant cells, whose morphology is extremely immature (Louis et al., 2016). GBMs show high invasiveness and proliferation rate. However, despite treatments, GBMs remain an incurable disease and the probability of survival at 5 years is of 9.8% in patients receiving concurrent chemo-radiotherapy and only 1.9% in those treated with radiotherapy alone (Stupp et al., 2005). Depending on specific genetic alterations, glioblastoma are distinct in primary and secondary subtypes with different phenotypic characteristics (Arcuri et al., 2017).

Mechanistic target of rapamycin (mTOR) is a 289-kDa serine/threonine kinase (Kunz et al., 1993) belonging to the phosphatidylinositol-4,5-bisphosphate 3-kinase (PI3K) family with homologs in all eukaryotes. In mammalian cells mTOR exists in two different complexes known as mTOR complex 1 (mTORC1) and mTOR complex 2 (mTORC2). mTORC1 is activated by at least five cues: growth factors, stress, energy status, oxygen and amino acid concentration. The principal process controlled by mTORC1 is protein synthesis (Laplante and Sabatini, 2012) and cell growth induction via autophagy inhibition (Hosokawa et al., 2009). mTORC2 phosphorylates AKT on threonine 450 and on serine 473 (Sarbassov et al., 2005). Phosphorylation on serine 473 is induced by growth factors and hormones; this post-translational modification occurs upon recruitment of AKT to the membrane. Serine 473 phosphorylation allosterically activates AKT, increasing the activity of AKT downstream targets (Hresko and Mueckler, 2005). Furthermore, mTORC2 controls protein kinase C maturation and stability (Facchinetti et al., 2008) and a role in actin cytoskeleton reorganization has emerged in studies regarding cell migration and cancer metastasis (Sarbassov et al., 2004).

Similar to mTORC1, mTORC2 could have a role in protein synthesis. Indeed, mTORC2 directly interacts with the 60S large ribosome subunit, and rapamycin-insensitive companion of mammalian target of rapamycin (RICTOR) can form stable associations with the ribosomal proteins L23a and L26 that are positioned at the exit tunnel. The nature of this interaction supports the hypothesis that mTORC2 plays a role in co-translational processes or maturation of nascent polypeptides (Oh et al., 2010).

mTOR plays a pivotal role in cell growth and metabolism and for this reason it is reasonable to suppose the existence of an association between the mTOR pathway activity and cancer. However, mutations that targets *mTOR*, conferring its constitutive activation have been identified in a minority of human tumors (Sato et al., 2010). Despite this, upstream regulators and mTOR downstream targets are frequently altered in human tumors (De Benedetti and Graff, 2004; Sansal and Sellers, 2004; Stemke-Hale et al., 2008). A growing body of evidence suggests that mTORC2 is involved in cancer cell metabolism, i.e., Warburg effect induction (Wu et al., 2014).

Further studies demonstrated mTOR upregulation in subependymal giant cell astrocytomas. These tumors often occur in the context of Tuberous Sclerosis Complex (TSC), a genetic and multisystem disorder caused by *TSC1* and *TSC2* mutations; following *TSC1/2* mutations, this complex does not work properly, therefore mTORC1 is activated by high RHEB-GTP levels (Jóźwiak et al., 2015). More recently, AKT expression and phosphorylation and RICTOR and Ki-67 expression have been evaluated in 195 human astrocytomas of different malignancy degree and 30 healthy controls. This analysis revealed that AKT expression and phosphorylation increases with the histological grade and correlates with a worse overall survival in GBMs, while RICTOR is overexpressed in grade I and II astrocytomas and a shift to a nuclear localization has been demonstrated in GBMs (Alvarenga et al., 2017).

mTOR inhibitor rapamycin and analogs (rapalogs) have cytostatic rather than cytotoxic properties and several reasons for failure of rapalogs as chemotherapeutic drugs in GBM have been proposed. First of all, rapalogs are selective mTORC1 inhibitors and the inhibition of mTORC1 downstream targets is not complete (Choo et al., 2008). Another reason is the existence of a feedback mechanism activated by mTORC1 inhibition that stimulates mitogenic pathways. mTORC1 activates S6K1 that in turn promotes insulin receptor substrate (IRS) proteolysis; in normal condition IRS facilitates insulin and inulin growth factor receptor signaling to activate PI3K. Rapalogs block S6K1-dependent auto-inhibitory pathway, which results in PI3K activation and induction of mTOR inhibitor resistance (Harrington et al., 2004). Finally, S6K1 activation induces RICTOR phosphorylation that in turn inhibits mTORC2; mTORC1 rapalog-induced inhibition relieves RICTOR inhibition and triggers AKT activation (Julien et al., 2010).

In order to overcome the limitations emerged in clinical studies that had evaluated rapalog based therapies, a second generation of mTOR inhibitors has been developed. These inhibitors are referred to as ATP-competitive mTOR kinase inhibitors (TORKIs; Chiarini et al., 2015; Jhanwar-Uniyal et al., 2015). Since both *in vitro* and *in vivo* studies showed that mTORC2 plays a pivotal role in cancer growth and survival, targeting mTOR with TORKIs might be more efficacious than rapalogs because of AKT phosphorylation inhibition downstream of mTORC2 (Roper et al., 2011).

Among TORKIs, PP242 induces mitophagy followed by cell death in ERas-treated cells (Gordeev et al., 2015), inhibits adult T cell leukemia proliferation and AKT phosphorylation on serine 473, *in vitro*, and reduces tumor growth in a leukemia xenograft mouse model (Kawata et al., 2018). Moreover, the administration of PP242 in a GBM cell line overexpressing EGFRvIII, causes cytoskeletal changes that affect cell motility (Chantaravisoot et al., 2015), while in GBM-bearing mice, the combined administration of PP242 and a glutaminase inhibitor induces cell death and reduces tumor growth (Tanaka et al., 2015).

To analyze the role of the PTEN/PI3K/AKT/mTOR pathway in GBM biology, we used an *in vitro* model composed of four GBM cell lines (i.e., GL15, U87MG, U251 and U118MG) characterized by different genetic alterations. Additionally, being U87MG cells endowed with glioblastoma stem cells (GSCs), we investigated whether that signaling pathway might also have a role in the maintenance of the cell population responsible for GBM relapse. In order to understand the specific role of PI3K and mTORC1, we selectively targeted PI3K with wortmannin and mTORC1 with rapamycin. On the other hand, to study the role of mTORC2 we used the ATP competitive mTOR inhibitor PP242, that targets both mTORC1 and mTORC2, as no molecules have been produced to target mTORC2 only. Based on this assumption, we referred throughout to PP242 as to mTORC2 inhibitor, and we deduced the role of mTORC2 by comparing the effects of inhibition of mTORC1 and mTORC2, mediated by PP242, and mTORC1 inhibition obtained with rapamycin administration.

## Materials and Methods

### Cell Culture Conditions

The GL15 cell line, that is not commercially available, was obtained by Bocchini et al. (1993) and is characterized by the presence of 7–8 extra copied of chromosome 7 and loss of chromosome 10, del9p. The U87MG cell line was purchased from ATCC^®^ (HTB-14™) and harbors PTEN (c.209 + 1G>T), CDKN2A (c.1_471 del471) and CDKN2B (c.1_507 del507) genetic alterations. The U251 cell line was purchased from CLS Cell line services (300385) and harbors TP53 (c.818G>A, p.R273H), PTEN (c.723_724insTT p.E242fsX15) and CDKN2A (c.1_471 del 471) genetic alterations. The U118MG cell line was purchased from ATCC^®^ (HTB-15™) and harbors PTEN (c.1026 + 1G>T), TP53 (c.638G>A) and CDKN2A (c.1_471del471) genetic alterations. All the cell lines were cultured in Dulbecco’s modified Eagle’s medium (DMEM; EuroClone^®^) supplemented with 10% fetal bovine serum (FBS; EuroClone^®^), 100 U/mL penicillin, 100 μg/mL streptomycin and 2.5 mM L-glutamine (EuroClone^®^). To induce GSC growth, U87MG cells were cultured in DMEM: Nutrient Mixture F-12 (DMEM-F12; Gibco^®^) supplemented with 100 U/mL penicillin and 100 μg/mL streptomycin (EuroClone^®^). All cell lines were grown in an H_2_O-saturated 5% CO_2_ atmosphere at 37°C.

### Chemical Reagents

PP242, wortmannin and rapamycin were purchased from Sigma Aldrich^®^ and dissolved in DMSO. The concentrations utilized in this study, established in preliminary experiments, were 2.5 μM PP242, 500 nM wortmannin and 1 μM rapamycin. Control cells received equal amounts of DMSO.

### Protein Analysis and Antibodies

Protein expression and phosphorylation levels were analyzed by immunoblotting. Cells were lysed with a lysis buffer containing 1M Tris-HCl, 20% SDS, 1 mM DTT, 200 mM PMSF, 10 mg/mL aprotinin (Gold Biotechnology, Olivette, MO, USA), 1 mg/mL pepstatin (EuroClone^®^) and 5 mg/mL leupeptin (Serva). Equal amounts of cell lysates were separated in 15%, 10% or 7.5% SDS-PAGE, depending on the antigen molecular weight. The following antibodies were used: monoclonal anti phosphorylated AKT (serine 473; 1:1000), polyclonal anti AKT (1:1000), polyclonal anti phosphorylated ERK1/2 (thr202/tyr204; 1:1000), polyclonal anti phosphorylated mTOR (serine 2448; 1:1000), polyclonal anti phosphorylated mTOR (serine 2481; 1:1000), polyclonal anti mTOR (1:1000), monoclonal anti LC3A/B (1:1000), monoclonal anti phosphorylated NF-kB p65 (serine 536; 1:1000; all from Cell Signaling Technology^®^), polyclonal anti NFkB p65 (1:1000), monoclonal anti α-tubulin (1:2000; Santa Cruz Biotechnology^®^), polyclonal anti MAP kinase (ERK1/2; 1:1000), goat anti rabbit IgG HRP (1:2000; Sigma Aldrich^®^) and goat anti mouse IgG/IgM HRP (1:5000; EMD Millipore). The immune reaction was developed by SuperSignal West Pico Luminol/Enhancer Solution and SuperSignal West Pico Stable Peroxide Solution (Thermo Fisher Scientific). Filters were subjected to densitometric analysis of the pertinent immune bands and their relative standard references using the software Image Studio Digit.

### Cell Viability Assay

Four-thousand cells were seeded in 96 well plates and treated with 2.5 μM PP242, 500 nM wortmannin or 1 μM rapamycin. Twenty-four, 48 h and 72 h after treatment, the cells were incubated with 3-(4,5-Dimethylthiazol-2-yl)-2,5-diphenyltetrazolium bromide (MTT) solution for 4 h. MTT powder was purchased from Sigma Aldrich^®^, dissolved in DMEM (1 mg/mL), filtered with 0.2 μm filter and stored for a few days at 4°C in the dark. After this incubation period, a water-insoluble formazan dye was formed. Following solubilization, the formazan dye was quantitated using a scanning multi-well spectrophotometer (ELISA reader). To verify cell growth a standard curve (from 1 × 10^1^ to 1 × 10^6^) was added in each plate.

### Migration and Invasion Assay

GL15, U87MG and U251 cells were seeded into six well plates and grown to confluence. The surface was scratched as uniformly as possible with a pipette tip to generate a wound. Detached cells were removed through a gentle wash with DMEM and the culture medium was replaced with serum-free DMEM plus 2.5 μM PP242, 500 nM wortmannin or 1 μM rapamycin. The wound area were photographed once a day till the wound area of control cells was completely closed using an Olympus IX51 microscope with 4× magnification for GL15 cells and 10× magnification for U87MG and U251 cells. The size of the wound area was calculated at each time point using the open source software Image J (MRI_wound_healing_tool-6). For invasion assay GL15, U87MG and U251 cells were harvested with serum-free DMEM and 200 μl of cell suspension (5 × 10^4^ cells) were seeded into the upper chamber (Falcon^®^), whereas the lower chamber was filled with 800 μl culture medium supplemented with 10% FBS. Following a 24 h incubation at 37°C, the cells were fixed with ice-cold methanol for 10 min and stained with 0.5% in PBS crystal violet for 15 min at room temperature. Images were captured using an Olympus IX51 microscope with 10× magnification.

### Immunofluorescence

For F-actin staining 4 × 10^4^ cells were seeded, treated with 2.5 μM PP242, 500 nM wortmannin or 1 μM rapamycin and cultured for 3 days (U87MG) and 7 days (GL15 and U251). Cells were extensively washed with PBS, fixed at room temperature with 4% paraformaldehyde in PBS for 20 min, permeabilized with 0.1% Triton X-100 in PBS for 10 min and washed three times with PBS. After overnight incubation with blocking buffer (3% bovine serum albumin [BSA], 1% glycine in PBS), cells were incubated at room temperature with TRITC-phalloidin (1:250; Sigma Aldrich^®^) in PBS for 30 min, washed three times with PSB 0.1% Tween and two times with PBS, incubated with 4’,6-diamidino-2-phenylindole (DAPI; 2 μg/ml; Sigma Aldrich^®^) for 30 s washed two times with PBS and air dried. For LC3 detection 4 × 10^4^ cells were seeded, treated with 2.5 μM PP242, 500 nM wortmannin or 1 μM rapamycin and cultured for 24 h on glass coverslips. Cells were extensively washed with PBS, fixed at room temperature with cold methanol for 7 min, permeabilized with 0.1% Triton X-100 in PBS for 5 min and washed three times with PBS. After overnight incubation with blocking buffer (3% BSA, 1% glycine in PBS), cells were subjected to immunofluorescence using a monoclonal anti LC3A/B (1:50; Cell Signaling Technologies) and a goat anti-rabbit TRITC (1:50; Sigma Aldrich^®^) antibodies.

For Bromodeoxyuridine (BrdU) incorporation assay 4 × 10^4^ cells were seeded, treated with 2.5 μM PP242, 500 nM wortmannin or 1 μM rapamycin and cultured for 24 h or 72 h on glass coverslips. Ten micrometer BrdU (Sigma Aldrich^®^) was added to the culture medium 1 h before fixation with cold methanol at −20°C. Cells were permeabilized with 0.1% Triton X-100 in PBS for 5 min, incubated with 2N HCl for 30 min, washed three times with sodium borate buffer and once with PBS. Then, cells were subjected to immunofluorescence using a monoclonal anti-BrdU (1:50; Santa Cruz Biotechnology^®^) and Alexa fluor^®^ 488 donkey anti-mouse (1:50; Life Technologies) antibody. BrdU-positive cells and total cells, counterstained with DAPI, were counted and the BruU-positive cells/total cells ratio was calculated. For comparative analysis, pictures were taken at a constant exposure time and gain in the same experimental setting, and ten random fields/coverslip were photographed. Coverslips were mounted and the preparations were viewed in a DMRB Leica epimicroscope equipped with a digital camera.

### Cell Cycle Analysis

Cell cycle was analyzed by flow cytometry. One-hundred-thousand cells were seeded into six well plates and treated with 2.5 μM PP242, 500 nM wortmannin or 1 μM rapamycin. Twenty-four hours and 48 h later, the culture medium of each sample was collected and centrifuged (400× *g*, 7 min) in order to recover apoptotic cells. Attached cells were washed twice with PBS and resuspended with 0.5 ml of hypotonic propidium iodide solution (50 μg/ml propidium iodide in 0.1% sodium citrate plus 0.1% Triton X-100) in 12 × 75 mm polypropylene tubes (BD Biosciences). The tubes were kept at 4°C for at least 30 min before flow cytometry analysis. Flow cytometry experiments were performed using Coulter Epics XL-MCL Flow Cytometer (Beckman Coulter) and data were analyzed using FlowJo software (TreeStar).

### RNA Extraction and Real Time PCR

Total RNA was extracted using the TRIsure™ reagent [BIOLINE] according to the manufacturer’s instructions and reverse-transcribed with PrimeScript RT Reagent Kit with gDNA Eraser [TaKaRa], prior to Real-Time PCR analysis using the primers specified below. Real-time PCR analysis was performed on a Stratagene Mx3000P (Agilent Technologies) using HOT FIREPol EvaGreen qPCR Mix Plus (ROX) ready-to-use solution [Solis BioDyne]. Amplification-curve plotting and calculation of Ct values were performed by a dedicated software.

Primer: NCBI Reference Sequence, Forward (5′-3′), Reverse (5′-3′). Human β-ACTIN (NM 001101.4) TCACCCACACTCTGCCCATCTACGA, CAGCGGAACCGCTCATTGCCAATGC. Human SOX2 (NM 003106) GCACATGAACGGCTGGAGCAACG, GCTGCGAGTAGGACATGCTGTAGG. Human OCT4 (NM 002701.5) TATTCAGCCAAACGACCATCT, TCAGCTTCCTCCACCCACTT.

### Statistical Analysis

Each experiment was performed at least three times and data are expressed as mean values ± SEM. Data were subjected variance (ANOVA) analysis using a statistical software package (GraphPad Prism, version 7.00).

## Results

### PP242 Inhibits mTOR Phosphorylation on Serine2481 and Serine2448

**Figure 1:**
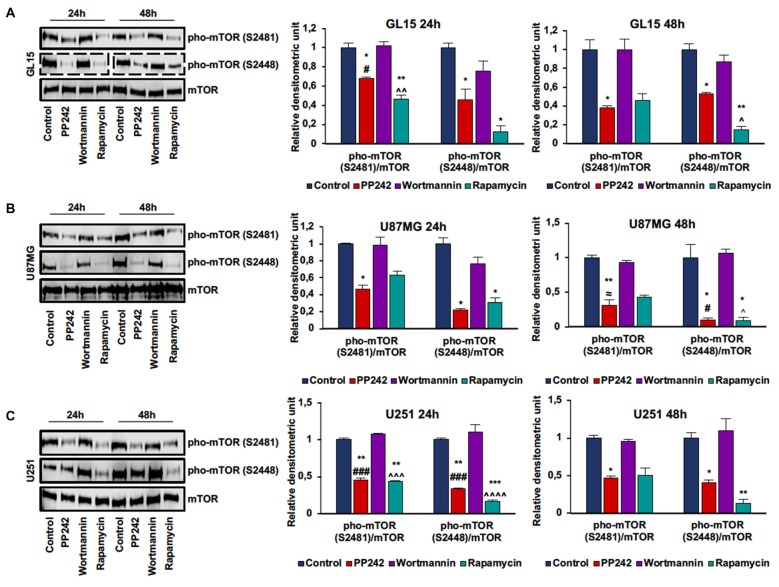
PP242 inhibits mechanistic target of rapamycin (mTOR) phosphorylation on serine2481 and serine2448. Western blots and densitometric quantification of mTOR phosphorylation on serine 2481 and serine 2448 in GL15 **(A)** U87MG **(B)** and U251 **(C)** cells. The cells were treated with 2.5 μM PP242, 500 nM wortmannin or 1 μM rapamycin for 24 h and 48 h, respectively. Blots are representative of at least three experiments and are expressed as mean values ± SEM. Legend: *Any inhibitor/control, ^#^PP242/wortmannin, ^≈^PP242/rapamycin, ^∧^rapamycin/wortmannin (^*,#,∧,≈^*p* < 0.05, ^**,∧∧^*p* < 0.01, ^***,###,∧∧∧^*p* < 0.001, ^∧∧∧∧^*p* < 0.0001).

In order to evaluate mTORC1 and mTORC2 activation, we analyzed mTOR phosphorylation on ser-2448 and ser-2481. Phosphorylation on ser-2448 is considered predictive of mTORC1 activation (Navé et al., 1999), whereas phosphorylation on ser-2481 serves as a biomarker for intact mTORC2 and its sensitivity to rapamycin (Copp et al., 2009). The irreversible inhibition of PI3K, obtained by the administration of wortmannin (Powis et al., 1994), did not modify either mTORC1 or mTORC2 activation in the cell lines considered as evidenced by the unchanged phosphorylation levels compared to the control even after 48 h of treatment (Figures 1A–C; Supplementary Figure S1A). In GL15 (Figure 1A), U251 (Figure 1C) and U118MG cells (Supplementary Figure S1), mTORC1 blockade with rapamycin significantly decreased mTOR phosphorylation on ser-2448 and ser-2481 after 24 h of treatment; however, only reduction of ser-2448 phosphorylation levels was retained after 48 h. Instead, in U87MG cells (Figure 1B) treatment with rapamycin significantly affected phosphorylation on ser-2448 only, even after 48 h of treatment. Contrariwise, the administration of PP242 for 24 h significantly decreased mTOR phosphorylation on ser-2448 and ser-2481 in the cell lines analyzed (Figures 1A–C, Supplementary Figure S1A). By extending the treatment to 48 h, this reduction of phosphorylation was maintained unchanged in GL15 (Figure 1A), U251 (Figure 1C) and U118 (Supplementary Figure S1A) cells and was further incremented in U87MG (Figure 1B) cells in which phosphorylation on ser-2448 and ser-2481 was reduced by more than 90% and 70%, respectively.

### PP242 Reduces Cell Viability and Proliferation

**Figure 2:**
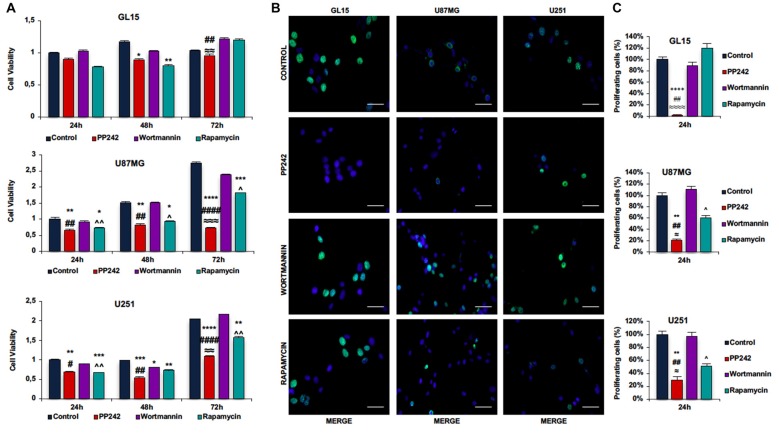
PP242 reduces cell viability and proliferation. Cell viability and proliferation assays. GL15, U87MG and U251 cells **(A)** were treated with 2.5 μM PP242, 500 nM wortmannin or 1 μM rapamycin for 24 h, 48 h and 72 h and 3-(4,5-Dimethylthiazol-2-yl)-2,5-diphenyltetrazolium bromide (MTT) assays were performed. Bromodeoxyuridine (BrdU; green) and 4′,6-diamidino-2-phenylindole (DAPI; blue) immunofluorescence of GL15, U87MG and U251 cells **(B)** treated with 2.5 μM PP242, 500 nM wortmannin or 1 μM rapamycin for 24 h (bar = 50 μm); the number of BrdU positive cells and total cells were counted and the BrdU positive cells/total cells ratio was calculated **(C)**. Data are shown as mean values ± SEM. Legend: *Any inhibitor/control, ^#^PP242/wortmannin, ^≈^PP242/rapamycin, ^∧^rapamycin/wortmannin (^*,#,∧,≈^*p* < 0.05, ^**,##,∧∧,≈≈^*p* < 0.01, ^***,≈≈≈^*p* < 0.001, ^****,####,≈≈≈≈^*p* < 0.0001).

To understand the role of the PTEN/PI3K/AKT/mTOR pathway in GBM cell viability and proliferation, we selectively inhibited PI3K, mTORC1 and mTORC2 in GL15, U87MG, U251 and U118 cells and performed MTT and BrdU incorporation assays. The irreversible inhibition of PI3K with wortmannin did not modify either cell viability or the number of BrdU-positive cells in the GBM cell lines analyzed (Figures 2A–C, Supplementary Figures S1B,C). Similarly, the blockade of mTORC1 with rapamycin did not change cell viability and the number of BrdU-positive cells in GL15 cells (Figures 2A–C); indeed, although cell viability decreased after 48 h, this reduction was not maintained after 72 h of treatment (Figure 2A). In U87MG cells, the inhibition of mTORC1 with rapamycin weakly decreased cell viability, reaching a 33% reduction after 72 h of treatment. Also, the number of BrdU-positive cells diminished but this reduction was statistically significant only if compared with wortmannin-treated cells (Figures 2A–C). A similar trend emerged in U251 cells. After treatment with rapamycin, cell viability slightly decreased, reaching about 23% reduction after 72 h of treatment; consistently, the number of BrdU-positive cells was lower, but only if compared with wortmannin-treated cells (Figures 2A–C). Instead, in U118 cells the inhibition of mTORC1 reduced cell viability without affecting cell proliferation (Supplementary Figures S1B,C). Contrariwise, in GL15 cells the inhibition of mTORC2 with PP242 decreased the number of BrdU-positive cells by more than 90%, as compared with control and wortmannin/rapamycin-treated cells; consistently, we observed that cell viability progressively decreased after 72 h of treatment (Figures 2A–C). In U87MG cells, blocking mTORC2 with PP242 resulted in an 80% decrease in the number of BrdU-positive cells and cell viability was progressively reduced up to 74% after 72 h of treatment (Figures 2A–C). Similarly, in U251 cells, mTORC2 inhibition reduced the number of BrdU-positive cells by 70%, while cell viability progressively decreased to 47% after 72 h (Figures 2A–C). In U118 cells the inhibition of mTORC2 decreased the number of BrdU-positive cells and cell viability by more than 50% (Supplementary Figures S1A,B).

**Figure 3:**
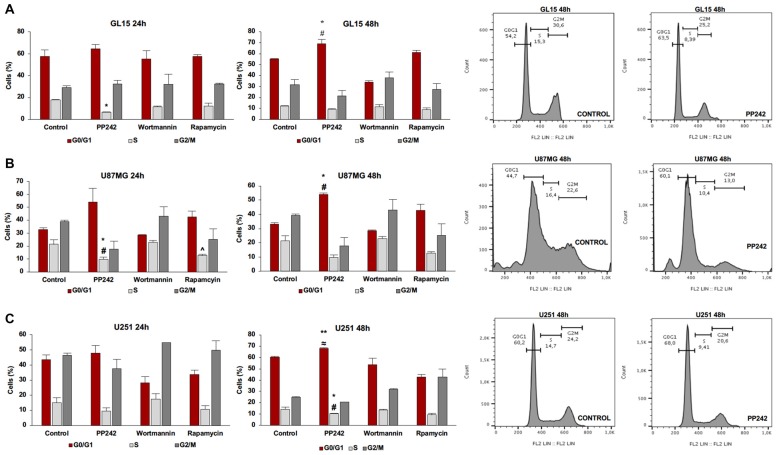
Cell cycle analysis. GL15 **(A)**, U87MG **(B)** and U251 **(C)** cells were treated with 2.5 μM PP242, 500 nM wortmannin or 1 μM rapamycin for 24 h and 48 h respectively. Cell distribution in G0/G1, S and G2/M phases was analyzed by flow cytometry using propidium iodide DNA staining. Legend: *Any inhibitor/control, ^#^PP242/wortmannin, ^≈^PP242/rapamycin, ^∧^rapamycin/wortmannin (^*,#,∧,≈^*p* < 0.05, **p < 0.01).

Having found that PI3K, mTORC1 and mTORC2 differently modulate cell proliferation, we analyzed the cell cycle distribution of GL15, U87MG and U251 cells treated with wortmannin, rapamycin or PP242. Coherently with data obtained by BrdU incorporation assay, the cell cycle analysis revealed that PI3K inhibition with wortmannin and mTORC1 blockade with rapamycin did not modify the percentage of cell distribution in G0/G1, S and G2/M phases in the three GBM cell lines (Figures 3A–C). Instead, in accordance with the aforementioned data, GL15 and U87MG cells treated with PP242 for 24 h, showed a reduced percentage of cells in S phase (Figures 3A,B) whereas in U251 cells, a similar trend of cell distribution was observed but it resulted not to be statistically significant (Figure 3C). After 48 h of treatment, the cell lines considered showed an increased percentage of cells in G0/G1 phase and U251 cells also revealed a significant reduction of the number of cells in S phase at this time point (Figures 3A–C).

### PP242 Reduces AKT and p65 Phosphorylation Without Affecting ERK1/2 Phosphorylation

To further understand the molecular mechanisms behind the reduction of cell proliferation triggered by mTORC2 inhibition, we analyzed AKT phosphorylation on ser-473, which represents the main target of mTORC2, and ERK1/2 phosphorylation on threonine 202/tyrosine 204 because this kinase is frequently phosphorylated in tumor cells when pro-survival stimuli decrease (Mendoza et al., 2011).

**Figure 4:**
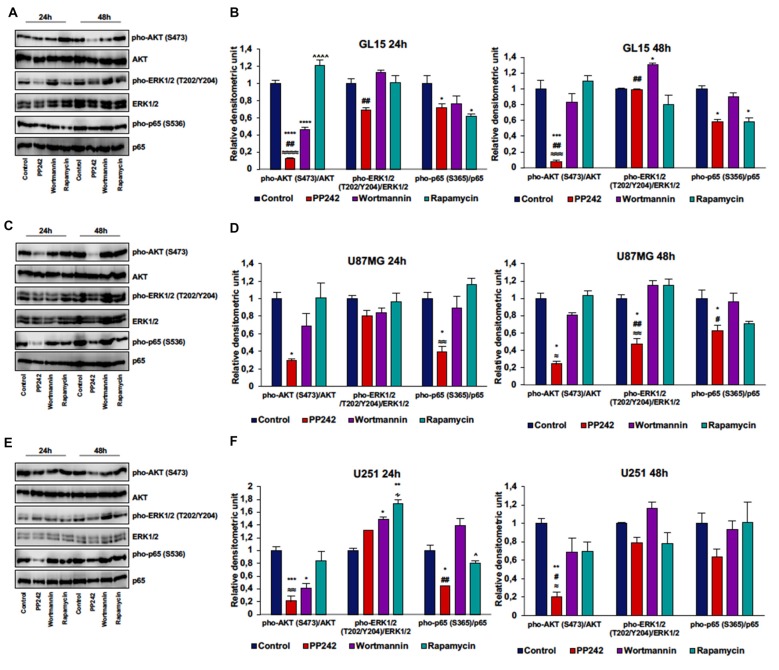
PP242 reduces AKT and NF-κB (p65) phosphorylation without affecting ERK1/2 phosphorylation. Western blot analysis of phosphorylated-AKT (S473), phosphorylated-ERK1/2 (T202/Y204) and phosphorylated-NF-κB (p65; S536) in GL15, U87MG and U251 cells **(A,C,E)** treated with 2.5 μM PP242, 500 nM wortmannin or 1 μM rapamycin for 24 h and 48 h, respectively. Densitometric analysis **(B,D,F)** of bands shown in **(A,C,E)**. Blots are representative of at least three experiments and values are expressed as mean ± SEM. Legend: *Any inhibitor/control, ^#^PP242/wortmannin, ^≈^PP242/rapamycin, ^∧^rapamycin/wortmannin ^∻^rapamycin/PP242 (^*,#,∧,≈,∻^*p* < 0.05, ^**,##,≈≈^*p* < 0.01, ^***,≈≈≈^*p* < 0.001, ^****,≈≈≈≈,∧∧∧∧^*p* < 0.0001).

Irreversible inhibition of PI3K with wortmannin reduced AKT phosphorylation on ser-473 after 24 h of treatment in GL15 and U251 cells but not in U87MG and U118 cells. However, after 48 h of treatment, AKT phosphorylation levels on ser-473 returned to high levels (Figures 4A–F, Supplementary Figure S1A).

In GL15 cells, the blockade of mTORC1 with rapamycin for 24 h did not reduce AKT phosphorylation on ser-473. Rather, rapamycin-treaded cells showed higher AKT phosphorylation levels on ser-473 compared to wortmannin- and PP242-treated cells and the difference between rapamycin-treated cells and PP242-treated cells was also present after 48 h of treatment (Figures 4A,B). Likewise, treatment with rapamycin did not significantly modify AKT phosphorylation, even after 48 h of treatment in both U87MG and U251 cells (Figures 4C–F). In U118 cells the inhibition of mTORC1 did not affect AKT phosphorylation on ser-473 after 24 h of treatment, while after 48 h we obswerved a reduction of AKT phosphorylation level (Supplementary Figure S1A). Contrariwise, in all the cell lines analyzed, mTORC2 inhibition with PP242 led to a significant and durable reduction of AKT phosphorylation on ser-473; this reduction reached 90% in GL15 cells and 80% in U87MG and U251 cells after 48 h of treatment (Figures 4A–F, Supplementary Figure S1A). Also, PI3K inhibition was accompanied by ERK1/2 phosphorylation in GL15 cells after 48 h of treatment and in U251 cells after 24 h, whereas in U87MG cells no significant changes were found (Figures 4A–F).

Inhibition of mTORC1 with rapamycin did not modify ERK1/2 phosphorylation in the three cell lines (Figures 4A–F); indeed, although in U251 cells we found an increase in ERK1/2 phosphorylation after 24 h of treatment, this increase was reversed after 48 h (Figures 4E,F). Similarly, mTORC2 inhibition did not significantly affect ERK1/2 phosphorylation, although a reduction of ERK1/2 phosphorylation was observed in U87MG cells after 48 h of treatment (Figures 4C,D). Notably, PP242-treated cells showed significantly less ERK1/2 phosphorylation levels than did wortmannin-treated cells (Figures 4A–F).

Additionally, in light of recent data supporting a role of mTORC2 in the induction of NF-κB(p65) phosphorylation as a chemotherapy resistance mechanism in GBM (Tanaka et al., 2011), we evaluated weather PI3K, mTORC1 or mTORC2 pharmacological inhibition could modulate NF-κB(p65) phosphorylation. We found that the irreversible inhibition of PI3K with wortmannin had no effects on NF-κB(p65) phosphorylation, while mTORC1 blockade with rapamycin only reduced NF-κB(p65) phosphorylation levels in GL15 cells (Figures 4A–F). Contrariwise, inhibition of mTORC2 with PP242 caused a significant decrease in NF-κB(p65) phosphorylation levels in the three cell lines and this inhibition persisted over the time (Figures 4A,F).

### PP242 Induces High Autophagy Levels

It is widely recognized that GBM cells are refractory to apoptosis induction. On the basis of this assumption, we investigated whether the reduction of cell viability and proliferation triggered by treatment with PP242, might result in the activation of the autophagy pathway as an alternative cell death mechanism.

Autophagy is induced by several cues including nutrient and growth factors availability, energetic status, hypoxia, oxidative stress and pathogen infection; these stress signals are integrated by mTOR that, under nutrient rich conditions, acts as a negative upstream regulator (Rubinsztein et al., 2012). However, the role of autophagy in cancer is controversial because, although autophagy is suppressed during tumor development, this pathway is upregulated during tumor progression, probably as a protective mechanism against stressful conditions (Choi, 2012). On the other hand, in cancer cells with a high apoptotic threshold, autophagy induction has emerged as a promising strategy to induce cell death (Gozuacik and Kimchi, 2004).

**Figure 5:**
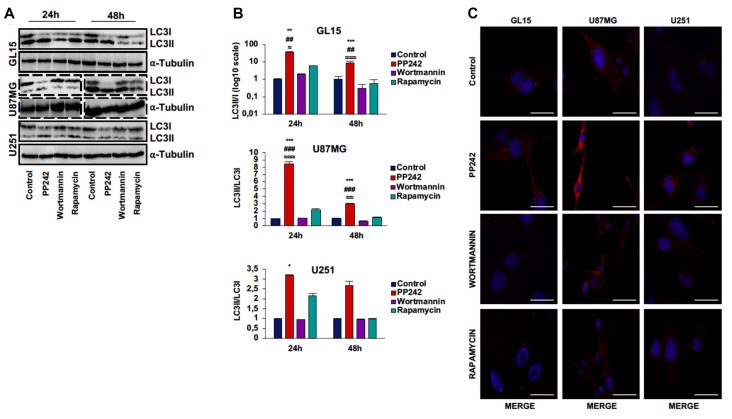
PP242 induces high autophagy levels. Western blot analysis of LC3I, LC3II and α-tubulin in GL15, U87MG cells **(A)** treated with 2.5 μM PP242, 500 nM wortmannin or 1 μM rapamycin for 24 h and 48 h, respectively. Densitometric quantification **(B)** of band intensities showed in **(A)**. Blots are representative of at least three experiments and are expressed as mean values ± SEM. **(C)** LC3 immunofluorescence in GL15, U87MG and U251 cells treated with 2.5 μM PP242, 500 nM wortmannin or 1 μM rapamycin for 24 h (bar = 25 μm). Legend: *Any inhibitor/control, ^#^PP242/wortmannin, ^≈^PP242/rapamycin (^*,≈^*p* < 0.05, ^**,##,≈≈^*p* < 0.01, ^***,###,≈≈≈^*p* < 0.001).

We analyzed the protein expression of LC3, one main autophagy marker (Kimura et al., 2009), and we observed that the irreversible inhibition of PI3K with wortmannin did not modify the expression and localization of LC3 (Figures 5A–C). However, we only found the expression of the cytosolic-associated LC3 isoform after the blockade of mTORC1 with rapamycin (Figure 5C). Instead, when we inhibited mTORC2 with PP242, we found that after 24 h of treatment, the expression of the cytosolic LC3I isoform was completely converted in the autophagosome-associated LC3II isoform in the three GBM cell lines (Figures 5A,B). The conversion of LC3I into LC3II was confirmed in PP242 treated-cells as evidenced by the increased number and size of LC3 positive dots detected by immunofluorescence staining (Figure 5C). A similar pattern of LC3II expression emerged in U118 cells as suggested by the increased number and size of LC3-positive dots in PP242-treated cells (Supplementary Figure S1D).

### PP242 Impairs Stem Cell Properties of U87MG Cells

**Figure 6:**
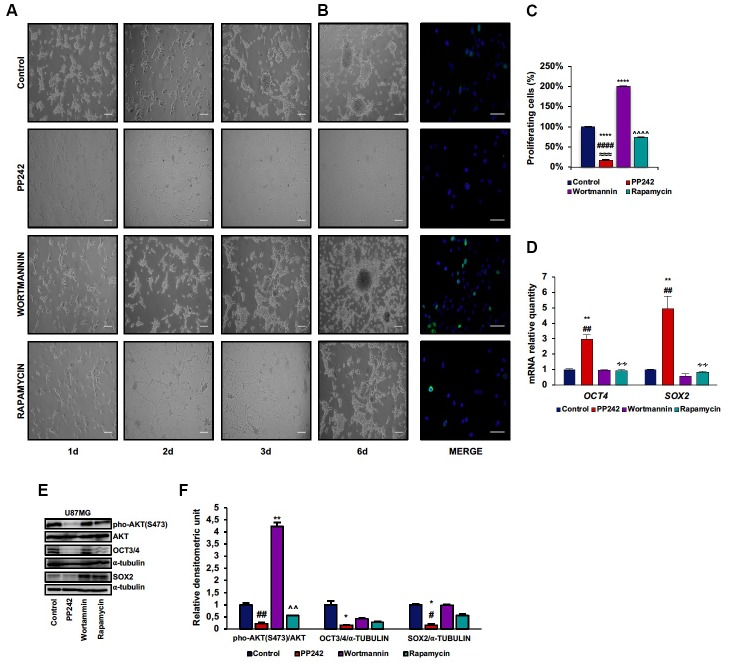
PP242 impairs stem cell properties of U87MG cells. Phase-contrast microscopy images showing neurospheres formation in U87MG cells **(A)** cultured in Dulbecco’s Modified Eagle Medium: Nutrient Mixture F-12 (DMEMF12) medium and treated with 2.5 μM PP242, 500 nM wortmannin or 1 μM rapamycin for 6 days (bar = 100 μm). BrdU (green) and DAPI (blue) immunofluorescence of U87MG cells **(B)** cultured in DMEMF12 medium and treated with 2.5 μM PP242, 500 nM wortmannin or 1 μM rapamycin for 72 h (bar = 50 μm). The number of BrdU positive cells and total cells **(C)** were counted and the BrdU positive/total cells ratio was calculated. Data are shown as mean values ± SEM. Relative mRNA expression of OCT4 and SOX2; U87MG cells **(D)** were cultured in DMEMF12 and treated with 2.5 μM PP242, 500 nM wortmannin or 1 μM rapamycin for 3 days. mRNA expression level was evaluated by Real Time PCR. Western blots of phosphorylated-AKT (serine 473), OCT4 and SOX2 in U87MG cells **(E)** cultured in DMEMF12 medium and treated with 2.5 μM PP242, 500 nM wortmannin or 1 μM rapamycin for 4 days. Densitometric analysis **(F)** of band shown in **(D)**. Blots are representative of at least three experiments and are expressed as mean values ± SEM. Legend: *Any inhibitor/control, ^#^PP242/wortmannin, ^≈^PP242/rapamycin, ^∧^rapamycin/wortmannin ^∻^rapamycin/PP242 (^*,#^*p* < 0.05, ^**,##,∧∧,∻∻^*p* < 0.01, ^≈≈≈^*p* < 0.001, ^****,####,∧∧∧∧^*p* < 0.0001).

U87MG is a heterogeneous cell line in which GSCs and more differentiated cells coexist (Vacas-Oleas et al., 2013). By cultivating these cells in DMEM-F12 medium it is possible to elicit GSC growth which can be phenotypically evaluated through the formation of free floating neurospheres. GSCs show cancer stem cell properties, including self-renewal, sphere forming ability, high proliferative rate and multipotency (Singh et al., 2004). Since the involvement of mTOR pathway in stem cell maintenance has recently emerged (Castilho et al., 2009; Easley et al., 2010), we investigated the role of PP242, wortmannin and rapamycin in this process. By monitoring cell morphology every day by phase-contrast microscopy, we observed that, as expected, control cells grew in clusters and that neurospheres began to develop at day 3 (Figure 6A). Unexpectedly, similar to control cells, wortmannin-treated cells rapidly aggregated in clusters, leading to neurosphere formation at day 3; furthermore, neurospheres continued to increase their diameter till day 6 (Figure 6A). Rapamycin-treated cells also tended to aggregate and grow in clusters yet without floating neurospheres (Figure 6A). Instead, cells treated with PP242 did not show any tendency to aggregate and lost the ability to form neurospheres. Moreover, we found a drastic morphological change consisting of cytoplasm retraction and appearance of numerous cell branches (Figure 6A). Having found that neurosphere formation began after 3 days in this culture, we analyzed cell proliferation at this time point. While mTORC1 inhibition did not affect cell proliferation, the number of BrdU-positive cells doubled after PI3K irreversible inhibition (Figures 6B,C). Contrariwise, mTORC2 inhibition caused a significant decrease in the number of proliferating cells (Figures 6B,C). Because mTORC2 inhibition prevented GSC proliferation and growth (Figures 6B,C), we analyzed the relative mRNA expression of the stemness markers *OCT4* and *SOX2* and surprisingly we observed a substantial upregulation of *OCT4* and *SOX2* expression after PP242 treatment only (Figure 6D). However, when we analyzed the protein expression of these transcription factors, we found that mTORC2 blockade resulted in a marked reduction of both OCT4 and SOX2 levels, whereas PI3K and mTORC1 inhibition did not influence their expression levels (Figures 6E,F).

### PP242 Modulates Actin Organization and Impairs Cell Migration and Invasiveness

**Figure 7:**
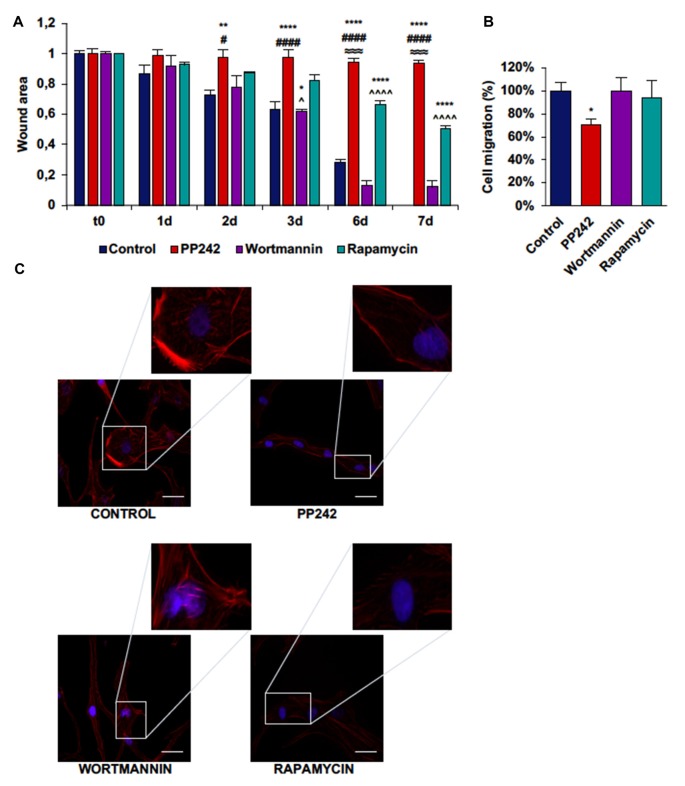
PP242 modulates actin organization and impairs cell migration and invasiveness of GL15 cells. Wound healing assay **(A)**. The wound areas were photographed and analyzed with Image J (MRI_wound_healing_tool-6). Transwell migration assay **(B)**. Migrated cells were stained with crystal violet and counted. Rhodamine-phalloidin (red) and DAPI (blue) immunofluorescence of GL15 cells **(C)** cultured for 7 days in serum-free DMEM plus 2.5 μM PP242, 500 nM wortmannin or 1 μM rapamycin (bar = 50 μm). Legend: *Any inhibitor/control, ^#^PP242/wortmannin, ^≈^PP242/rapamycin, ^∧^rapamycin/wortmannin (^*,#,∧^*p* < 0.05, **p < 0.01, ^≈≈≈^*p* < 0.001, ^****,####,∧∧∧∧^*p* < 0.0001).

Next, we performed a scratch assay to analyze the involvement of PI3K, mTORC1 and mTORC2 in GBM cell migration. In order to avoid filling up of the wound by proliferating rather than migrating cells, these tests were conducted under non-proliferative conditions. Control GL15 cells showed a high migration rate. These cells began to close the wound area 1 day after the scratch at a rate of 10%/day; wound closure proceeded at this rate until day 3 when the migration rate became faster. At day 7 the wound was completely closed (Supplementary Figure S2A). The irreversible inhibition of PI3K with wortmannin did not modify the ability of these cells to close the wound as only approximately 10% of the area was open after 7 days (Figure 7A). Contrariwise, mTORC1 blockade with rapamycin significantly slowed the wound closure as 50% of the wounded area was still open at day 7 (Figure 7A). Remarkably, mTORC2 inhibition with PP424, completely inhibited cell migration; 7 days after treatment with PP242, more than 95% of the wound area was still open (Figure 7A). Notably, a reduction of directional cell migration emerged from transwell migration assay in cell treated with PP242 for 24 h but not in cells treated with wortmannin or rapamycin (Supplementary Figure S2B, Figure 7B).

To further understand how cell migration was differently modulated by PI3K, mTORC1 and mTORC2, we analyzed F-actin organization by rhodamine-phalloidin immunofluorescence. Rapamycin-treated cells and to a greater extent, PP242-treated cells showed actin stress fiber disassembly and lack of F-actin accumulation at the leading edge, while control and wortmannin-treated cells showed many and thick actin stress fibers and F-actin accumulation at the leading edge (Figure 7C).

**Figure 8:**
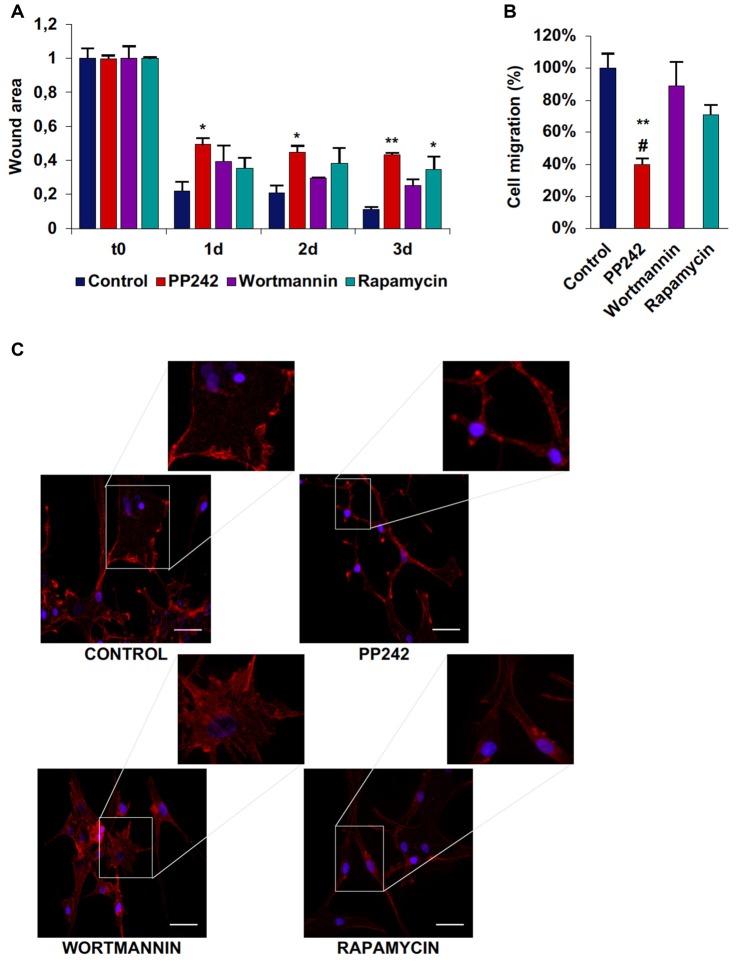
PP242 modulates actin organization and impairs cell migration and invasiveness of U87MG cells. Wound healing assay **(A)**. The wound areas were photographed and analyzed with Image J (MRI_wound_healing_tool-6). Transwell migration assay **(B)**. Migrated cells were stained with crystal violet and counted. Rhodamine-phalloidin (red) and DAPI (blue) immunofluorescence of U87MG cells **(C)** cultured for 3 days in serum-free DMEM plus 2.5 μM PP242, 500 nM wortmannin or 1 μM rapamycin (bar = 50 μm). Legend: *Any inhibitor/control, ^#^PP242/wortmannin (^*,#^*p* < 0.05, **p < 0.01).

Among the three cell lines analyzed, control U87MG cells showed the fastest migration rate in terms of wound healing; between time 0 and day 1 the wound was 75% closed and at day 3 only 10% of the wound area was still open (Supplementary Figure S3A, Figure 8A). Wortmannin and rapamycin-treated cells initially showed a similar pattern of migration as evidenced by a 65% wound closure in both samples at day 1, however starting from this time point, the wound remained unchanged till day 3 in rapamycin-treated cells, while it progressively decreased in wortmannin-treated cells (Figure 8A). Contrariwise, PP242-treated cells invaded the wound area more slowly than did control, wortmannin and rapamycin-treated cells as half of the initial area was open at day 1 and this value did not change till the last experimental time (Figure 8A). Consistently, transwell migration assay revealed that rapamycin reduced directional migration by 30% and PP242 decreased it by 60%, while wortmannin did not affect directional migration (Supplementary Figure S3B, Figure 8B).

As analyzed by F-actin organization, in U87MG cells F-actin did not form thick stress fibers, however, control and wortmannin-treated cells showed an extremely flat morphology characterized by the presence of F-actin accumulation at the leading edge. Instead, rapamycin- and to a greater extent, PP242-treated cells showed thin cytoplasm and numerous branches along which many focal adhesions could be seen (Figure 8C).

**Figure 9:**
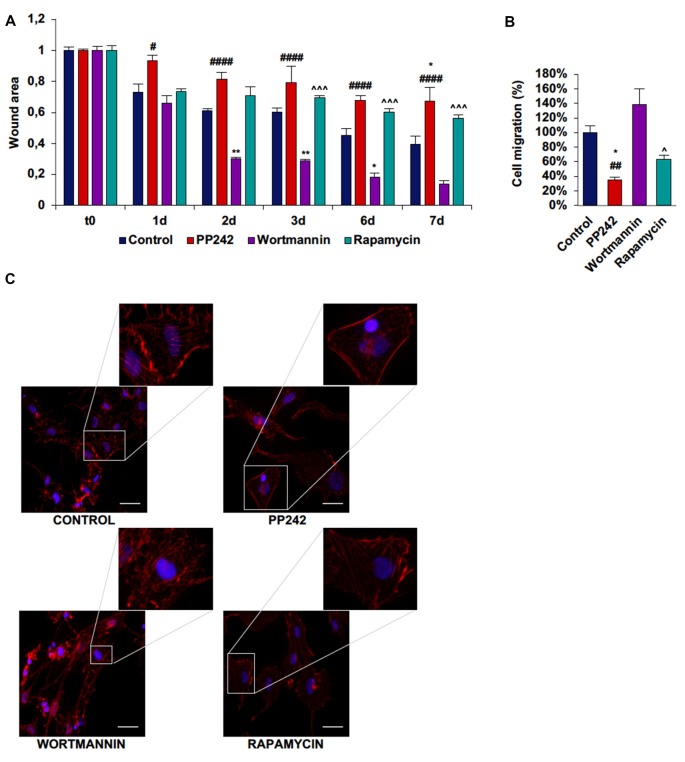
PP242 modulates actin organization and impairs cell migration and invasiveness of U251 cells. Wound healing assay **(A)**. The wound areas were photographed and analyzed with Image J (MRI_wound_healing_tool-6). Transwell migration assay **(B)**. Migrated cells were stained with crystal violet and counted. Rhodamine-phalloidin (red) and DAPI (blue) immunofluorescence of U251 cells **(C)** cultured for 3 days in serum-free DMEM plus 2.5 μM PP242, 500 nM wortmannin or 1 μM rapamycin (bar = 50 μm). Legend: *Any inhibitor/control, ^#^PP242/wortmannin, ^∧^rapamycin/wortmannin (^*,#,∧^*p* < 0.05, ^**,##^*p* < 0.01, ^∧∧∧^*p* < 0.001, ^####^*p* < 0.0001).

In U251 cells, control cells tended to close the wound area at a rate of 10%/day, but differently from GL15 cells, the migration rate did not become faster after day 3 (Supplementary Figure S4A; Figure 9A). Wortmannin-treated cells immediately invaded the wound; at day 1 about 35% of the area was already closed and the closure became 70% at day 2. At day 3 the migration rate slowed down and proceeded at a rate of about 10%/day till day 7 when about 87% of the area was closed (Figure 9A). Rapamycin-treated cells started to close the wound at the same rate as did wortmannin-treated cells, but the wound area remained almost unchanged till day 7 when more than half of the initial area was still open. Instead, PP242-treated cells showed the most static phenotype as the wound area was not modified during the experimental time and more than 66% of the wound was still free of cells after 7 days of treatment (Figure 9A). Similarly, transwell migration assay confirmed that wortmannin treatment did not affect directional migration, while rapamycin slightly reduced it. Instead, PP242 treatment significantly reduced directional cell migration by more than 60% (Supplementary Figure S4B, Figure 9B). Further, control cells showed numerous stress fibers arranged along the entire cellular surface and F-actin accumulation at the leading edge and an essentially similar picture could be observed in wortmannin-treated cells. Instead, rapamycin-treated cells showed a reduced number of stress fibers, and in PP242-treated cells stress fibers were completely destroyed (Figure 9C).

In U118MG, control cells invaded the wound area at a rate of about 20%–25%/day and, at day 3 more than half of the wound was closed by migrated cells (Supplementary Figure S1E). Wortmannin-treated cells started to close the wound more slowly than did control cells, but at day 3 we found the same wound closure as untreated cells. Rapamycin-treated cells closed the wound at a rate of about 10% at day 1 but thereafter the wound area remained almost unchanged. Contrariwise, the wound area of PP242-treated cells remained completely open till the last experimental time. Transwell migration assay confirmed the data of the wound healing assay, with the exception of rapamycin-treated cells which behaved much as untreated cells and wortmannin-treated cells in terms of directional migration (Supplementary Figure S1E).

## Discussion

GBM is an incurable disease because of its thorny localization and because the molecular mechanisms underlining its development and progression are far from being elucidated. The lack of knowledge of the complex network of interactions existing between the numerous pathways driving tumor growth and GSC proliferation has resulted into the failure of almost all clinical trials conducted to date. For this reason, in the light of accumulating evidence suggesting that the PTEN/PI3K/AKT/mTOR pathway is frequently deregulated in GBM and of recent data unveiling its involvement in cancer stem cell maintenance, we have focused our study on the kinase complex mTOR with the purpose to understand the role of mTORC2 in GBM biology compared with PI3K and mTORC1.

### PP242 Effectively Inhibits mTORC1 and mTORC2 Activation

Although PI3K is considered an mTOR upstream positive regulator, our data showed that, regardless of the *PTEN* status, the activation of mTOR appears to be independent of PI3K activation in GBM cells. These findings might explain why targeting PI3K is not a proper choice for GBM treatment and suggest the existence of a different, unknown signaling pathway that directly activates mTOR by bypassing PI3K downstream signaling. Furthermore, our data confirm that treatment with rapamycin is able to counteract mTORC1 activation and demonstrate that mTORC2 activation in GL15 and U251 cells but not in U87MG cells is rapamycin sensitive. However, while mTORC1 inhibition is long lasting, the ability of rapamycin to control mTORC2 activation decreases with time, suggesting that what we observed is an acute response and that the main rapamycin target is mTORC1 only. Contrariwise, the second-generation mTOR inhibitor, PP242, that interacts with the ATP binding site of mTOR, effectively blocks the activation of both mTORC1 and mTORC2, regardless of the *PTEN* status, in the GBM cell lines analyzed. Moreover, our data demonstrate that the inactivation of mTORC1 and mTORC2 persists over the time, suggesting that PP242 produces long-lasting effects.

### PP242 Reduces Cell Viability and Proliferation Decreasing AKT and NF-κB(p65) Phosphorylation, Without Affecting ERK1/2 Activation Status

To explore the role of PI3K, mTORC1 and mTORC2 in GBM biology, we initially evaluated the contribution of these kinases, taken individually, in cell viability and proliferation mechanisms. We found that the inhibition of PI3K, even if irreversible, has no impact on GBM metabolic activity; moreover, by reducing only transiently AKT phosphorylation, wortmannin does not affect the ability of GBM cells to cycle and proliferate, even in presence of wild type *PTEN* genotype. These data suggest that PI3K is not essential for AKT activation. We speculated that mTORC2 might be responsible for GBM cell proliferation, as its activation is sufficient to trigger AKT phosphorylation on ser-473. Indeed, regardless of the *PTEN* status, the inhibition of mTORC2 permanently reduces AKT phosphorylation on ser-473, which is necessary for full AKT activation. Lower AKT levels result in a significant reduction of cell proliferation and accumulation of cells in G0/G1 phase, besides a decreased metabolic activity. Furthermore, it is worth mentioning that mTORC2 blockade does not trigger ERK1/2 phosphorylation as a feedback mechanism to balance the lack of pro-survival stimuli, similar to other tumor cells (Mendoza et al., 2011; Ning et al., 2017).

Additionally, our data are in accordance with recent results showing that the activation of NFκB is mTORC2-dependent in GBM cells and that NF-κB(p65) phosphorylation is associated to the development of resistance to conventional chemotherapy (Tanaka et al., 2011). Consistently, we found that the irreversible inhibition of PI3K has no effects on NF-κB(p65) phosphorylation, while mTORC1 blockade reduces NF-κB(p65) phosphorylation only in cells with wild type *PTEN* genotype. On the other hand, the inhibition of mTORC2 permanently reduces NF-κB(p65) phosphorylation in GBM cells, regardless of the *PTEN* status. This suggests that targeting mTORC2 might be also useful to avoid the onset of resistance against the currently standard anti-neoplastic treatments. Instead, although acute (≤24 h) administration of rapamycin seems to affect mTORC2 activation, this does not result in any modification of cell proliferation and metabolic activity, as confirmed by the high level of AKT phosphorylation on ser-473 and cell cycle analysis. Together, these data suggest that rapamycin treatment is not sufficient to decrease mTORC2 levels below the threshold necessary to impact AKT phosphorylation.

### PP242 Induces High Autophagy Levels

Autophagy has an important role in tumor development (Lorin et al., 2013), and the constitutive activation of the PI3K/AKT/mTOR pathway is a hallmark of numerous tumors and has been reported to suppress the autophagy pathway (Wang et al., 2012). In the context of brain tumors, expression of *BECN1*, a specific autophagy gene, is lower in GBMs compared to lower grade astrocytomas and normal brain tissues. Moreover, the existence of a positive correlation between BECLIN1 expression and patient survival and performance status has been demonstrated (Miracco et al., 2007; Pirtoli et al., 2009). Additionally, high expression of LC3, another autophagy factor, is associated with improved survival in patients with poor performance scores, while in patients with normal scores it correlates with better survival. These data suggest that tumor progression could be triggered by decreased autophagy (Aoki et al., 2008). Interestingly, most genetic alterations harbored by brain tumors including alterations of *EGFR, NF1, PTEN* and *AKT*, are known to be involved in autophagy regulation. Several mechanisms have been proposed to explain autophagy-mediated tumorigenesis suppression, including autophagy-genomic stability maintenance and oncogene-induced senescence (Kaza et al., 2012). However, some authors assert that autophagy induction may be a mechanism of chemotherapy resistance (Chen and Karantza-Wadsworth, 2009; Chen et al., 2010).

Our data show that mTORC2 but not mTORC1 has a crucial role in the regulation of GBM cells growth, as its inhibition results in a dramatic reduction of cell proliferation and metabolic activity. However, GBM cells are particularly refractory to apoptosis induction as they frequently harbor *TP53* mutations. On the basis of these assumptions and considering that mTOR activation negatively controls autophagy, we investigate whether this pathway might be activated as an alternative cell death mechanism upon mTOR inhibition. As expected, the irreversible inhibition of PI3K did not induce the autophagy pathway, as it is widely proved that the generation of PIP3 is necessary for the autophagosome formation, and wortmannin is considered to act as an autophagy inhibitor (Ohsumi, 2001).

Surprisingly, we observed that the blockade of mTORC1 with rapamycin is not sufficient to induce autophagy, as evidenced by the balanced expression of the cytosolic and the autophagome-associated LC3 isoforms in the GBM cell lines analyzed. This observation, together with the induction of AKT phosphorylation triggered by intact mTORC2 and feedback mechanism following mTORC1 inhibition, might further explain why treatment of GBM patients with rapamycin analogs have failed. Contrariwise, the inhibition of mTORC2 with PP242 induces very high levels of autophagy already after 24 h of treatment in all the GBM cell lines considered and this process persists over time without further PP242 administration. These results suggest that in GBM, the induction of the autophagy pathway is dependent on mTORC2 but not mTORC1 and that mTORC2 inhibition might represent a reliable approach to trigger autophagy as an alternative cell death mechanism.

### PP242 Counteracts GSCs Self-Renewal

Taking into account that GBM is characterized by an extremely heterogeneous cell population and that GSCs are reasonably responsible for GBM recurrence, we were also interested to understand the impact of mTOR on GSCs, also in light of the recent observation that mTOR activation is responsible for the increased expression of a stem cell phenotype in tumor cells and that environmental cues might reprogram tumor cells in stem-like cells (Xia and Xu, 2015; Jhanwar-Uniyal et al., 2017). To this end, we cultured U87MG cells in conditions promoting promote GSCs growth phenotypically evaluable by the formation of free floating neurospheres. We found that the irreversible inhibition of PI3K with wortmannin further enhances GSC proliferation, as confirmed by the increased number of BrdU-positive cells, high AKT phosphorylation levels on ser-473 and well formed and free floating neurospheres. Instead, treatment with rapamycin has no effects on GSCs, because cell proliferation, AKT phosphorylation and neurosphere formation are comparable to those of control cells. Contrariwise, mTORC2 blockade not only dramatically reduces cell proliferation, as confirmed by the decreased AKT phosphorylation and BrdU incorporation; it also completely inhibits neurosphere formation. Intriguingly, we observed an increased mRNA expression of the stemness markers *OCT4* and *SOX2* in GSCs treated with PP242, which is only apparently at odds with this gene expression profile being a hallmark of cell senescence (Ritschka et al., 2017). Indeed, we observed a reduction of OCT4 and SOX2 protein levels after administration of PP242. Although this latter point needs to be elucidated, it is not unreasonable to suppose a post-transcriptional regulation of *OCT4* and *SOX2* expression which determines a reduced protein synthesis. However, a reduction of protein synthesis as an epiphenomenon of cellular senescence cannot be ruled out and this evidence should be considered as “hypothesis generating”. Together, our results strongly suggest that mTORC2 but not mTORC1 plays a pivotal role in GSC self-renewal and proliferation, suggesting that inhibiting mTORC2 might be a useful strategy to control GBM growth and to counteract GSC proliferation recognized responsible for GBM relapse (Huang et al., 2010).

### PP242 Modifies Actin Cytoskeleton Organization and Impairs Cell Migration and Invasiveness

GBM is a highly invasive tumor. Based on mTOR involvement in cytoskeleton organization (Sarbassov et al., 2004), we investigated the role of PI3K, mTORC1 and mTORC2 in the motility and invasiveness of cell lines harboring different genetic alterations. We found that PI3K does not participate in the control of cell migration, as its irreversible inhibition with wortmannin does not modify the ability of these tumor cells to invade and close a wound; surprisingly, the rate of the wound closure is even higher in wortmannin-treated as compared to control cells. Moreover, F-actin immunostaining revealed that, as in the case of control cells, wortmannin-treated cells display numerous and tick stress fibers on the entire cell surface, along with F-actin accumulation at the leading edge, supporting the idea that cell movement is tightly dependent on F-actin polymerization which is not affected by PI3K inhibition. Similarly, transwell migration assays confirmed that PI3K inhibition does not affect directional cell migration, while mTORC1 and to a greater extend mTORC2 blockade reduce it.

On the contrary, our data demonstrated that the kinase complex mTOR is crucial for cell motility regulation and that in this process the mTORC1 and mTORC2 contribution is not the same. Indeed, whereas in GL15 cells mTORC1 inhibition slows down the migration rate and reduces actin stress fiber number, mTORC2 inhibition completely arrests cell migration and abolishes actin stress fiber formation. In U87MG cells, that show the fastest migration rate, and in U251 cells, mTORC1 and mTORC2 inhibition reduces wound closure to a similar extent, though statistical analysis revealed that mTORC2 blockade is more efficacious than mTORC1. In U118 the mTORC1 and mTORC2 contribution in cell migration is the same as described for GL15 cells. Consistently, in GL15, U87MG and U251 cells F-actin organization analysis revealed that, while mTORC1 inhibition reduces the number and thickness of stress fibers, mTORC2 blockade completely impairs their formation and dramatically modifies cell morphology. These results suggest that the regulation of F-actin cytoskeleton organization and cell motility is tightly dependent on mTORC2 activation in GBM.

## Conclusion

GBM remains an incurable disease and intense efforts are still necessary to elucidate the biology of this tumor, with special attention to the complex network of signaling pathways that drive tumor growth, invasiveness and GSC maintenance. In this context, our *in vitro* study, which focused on the intricate PTEN/PI3K/AKT/mTOR pathway, revealed interesting insights that might explain the reason for failure of numerous clinical trials, and suggest a new potential target to counteract not only GBM growth and invasiveness but also GSCs self-renewal. Indeed, we found that, despite PTEN dysfunction, targeting PI3K has no impact on GBM cell proliferation and migration and even enhances GSC growth. Moreover, although PI3K is considered the only known mTOR upstream activator, its irreversible blockade does not prevent mTOR activation, suggesting that the activation of mTOR is independent of PI3K. Rather, a different and unknown upstream regulator might be responsible for mTOR activation. Further studies are warranted to address this issue.

We confirmed that mTORC1 inhibition by rapamycin administration does not represent an efficacious therapeutic strategy, as AKT phosphorylation triggered by mTORC1 inhibition via a feedback mechanism and the presence of an intact mTORC2 stimulate cell proliferation, with no effect on either apoptosis or autophagy. Conversely, mTORC2 blockade obtained by PP242 effectively inhibits the catalytic activity of both mTOR complexes, thus preventing the activation of feedback mechanisms induced by rapamycin and reducing cell proliferation. Moreover, PP242 prevents cell motility and contributes to limit the onset of chemotherapy resistance mechanisms via NF-κB(p65) phosphorylation inhibition.

Interestingly, our data show that the activation of autophagy in GBM is mTORC2- but not mTORC1-dependent and that the induction of the autophagy pathway at very high levels might represent a “trojan horse” for GBM cells, that are refractory to apoptosis. Finally, our preliminary results show that PP242 might prevent GSC self-renewal and growth. We propose that mTORC2 targeting might represent a valid choice for GBM treatment. However, we need to extend our analyses to human biopsies and to *in vivo* models to confirm our data.

## Author Contributions

CM performed the principle experiments and wrote the manuscript. SB and OB performed and interpreted cell cycle experiments. CR contributed to interpretation of data. IG and BF searched the literature, organized and created the figures and critically discussed the contents of the manuscript. RD and CA organized, reviewed and edited the manuscript.

## Conflict of Interest Statement

The authors declare that the research was conducted in the absence of any commercial or financial relationships that could be construed as a potential conflict of interest.

## Abbreviations

GBM: glioblastoma multiforme
GSCs: glioblastoma stem cells
IRS: insulin receptor substrate
mTOR: mechanistic target of rapamycin
mTORC1: mTOR complex 1
mTORC2: mTOR complex 2
PI3K: phosphatidylinositol-4,5-bisphosphate 3-kinase
RICTOR: Rapamycin-insensitive companion of mTOR
TORKIs: ATP-competitive mTOR kinase inhibitors
TSC: tuberous sclerosis complex.
