# Supplementary figures and images for: PP242 Counteracts Glioblastoma Cell Proliferation, Migration, Invasiveness and Stemness Properties by Inhibiting mTORC2/AKT
Source: Front Cell Neurosci. 2018 Apr 10;12:99. doi: 10.3389/fncel.2018.00099 (PMC5902688; doi:10.3389/fncel.2018.00099)

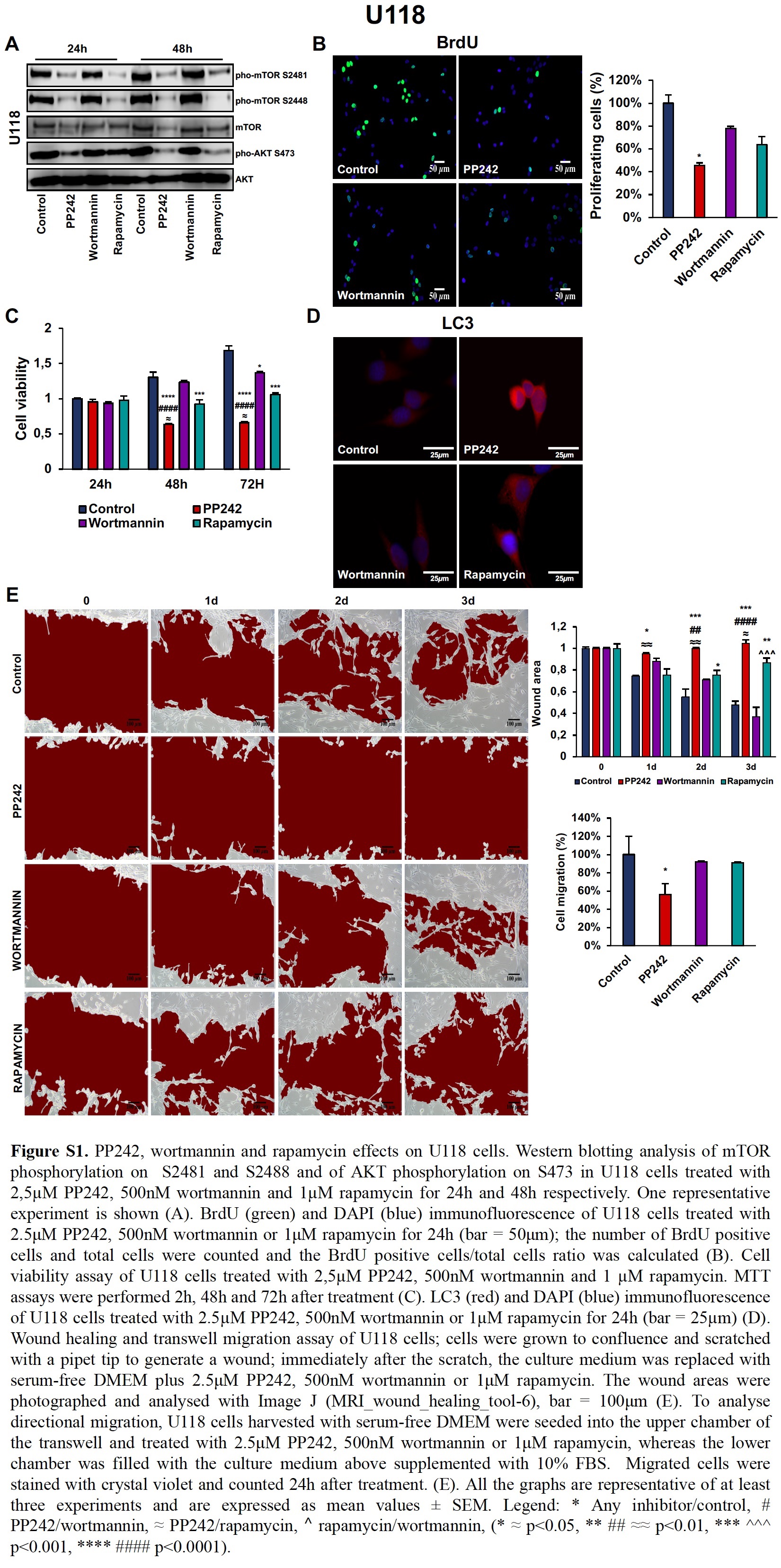

Supplement: Supplementary file 1 [file Image_1.jpg]

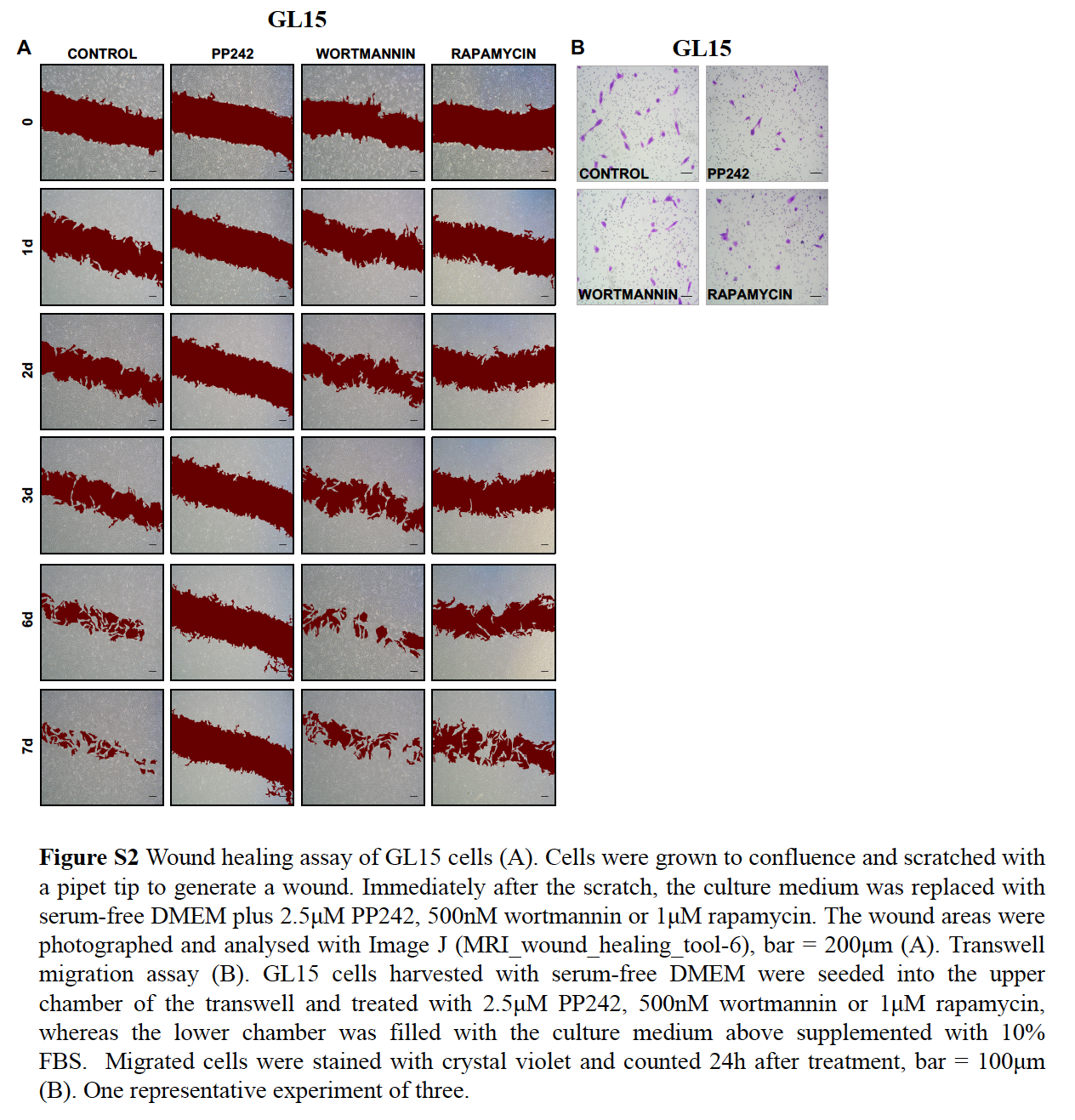

Supplement: Supplementary file 2 [file Image_2.tif]

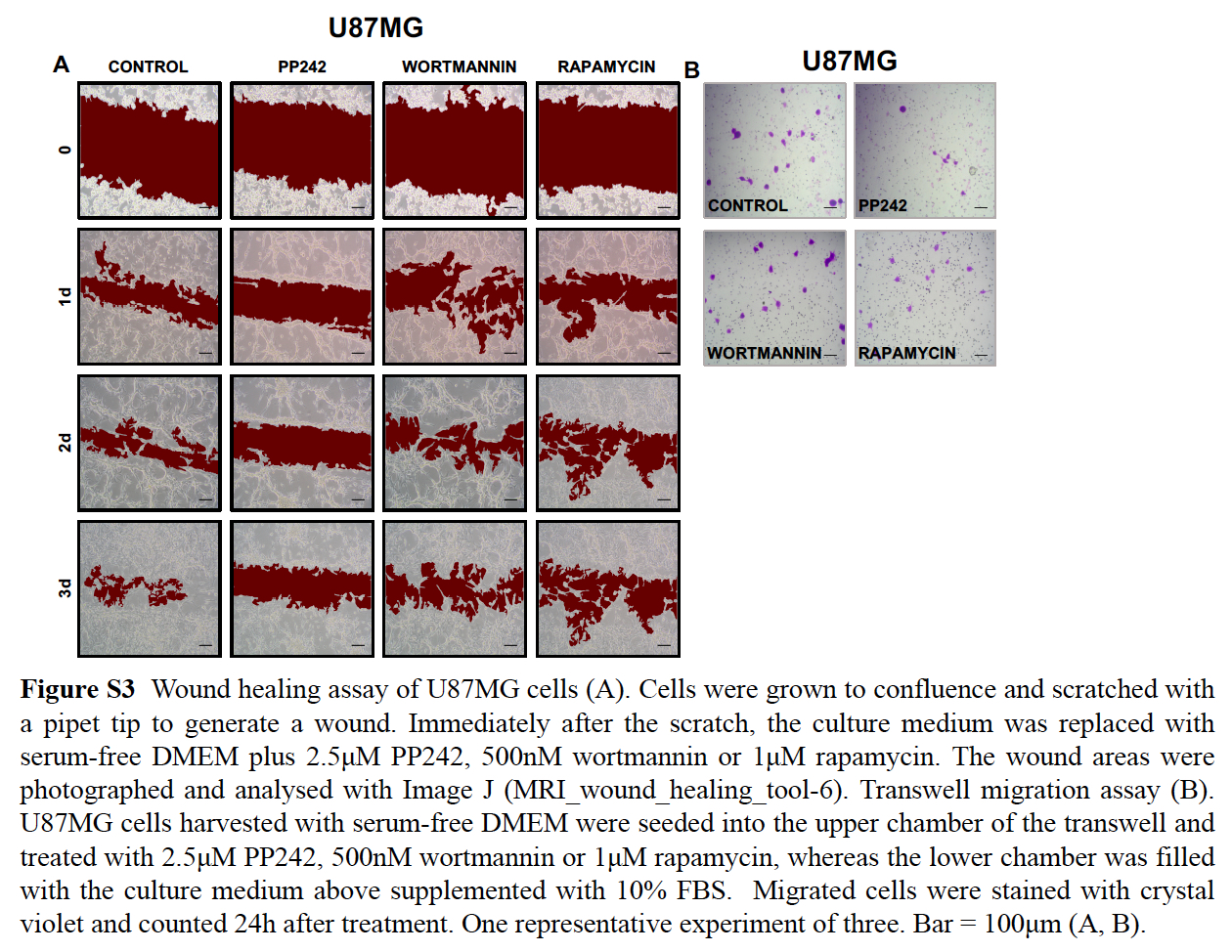

Supplement: Supplementary file 3 [file Image_3.tif]

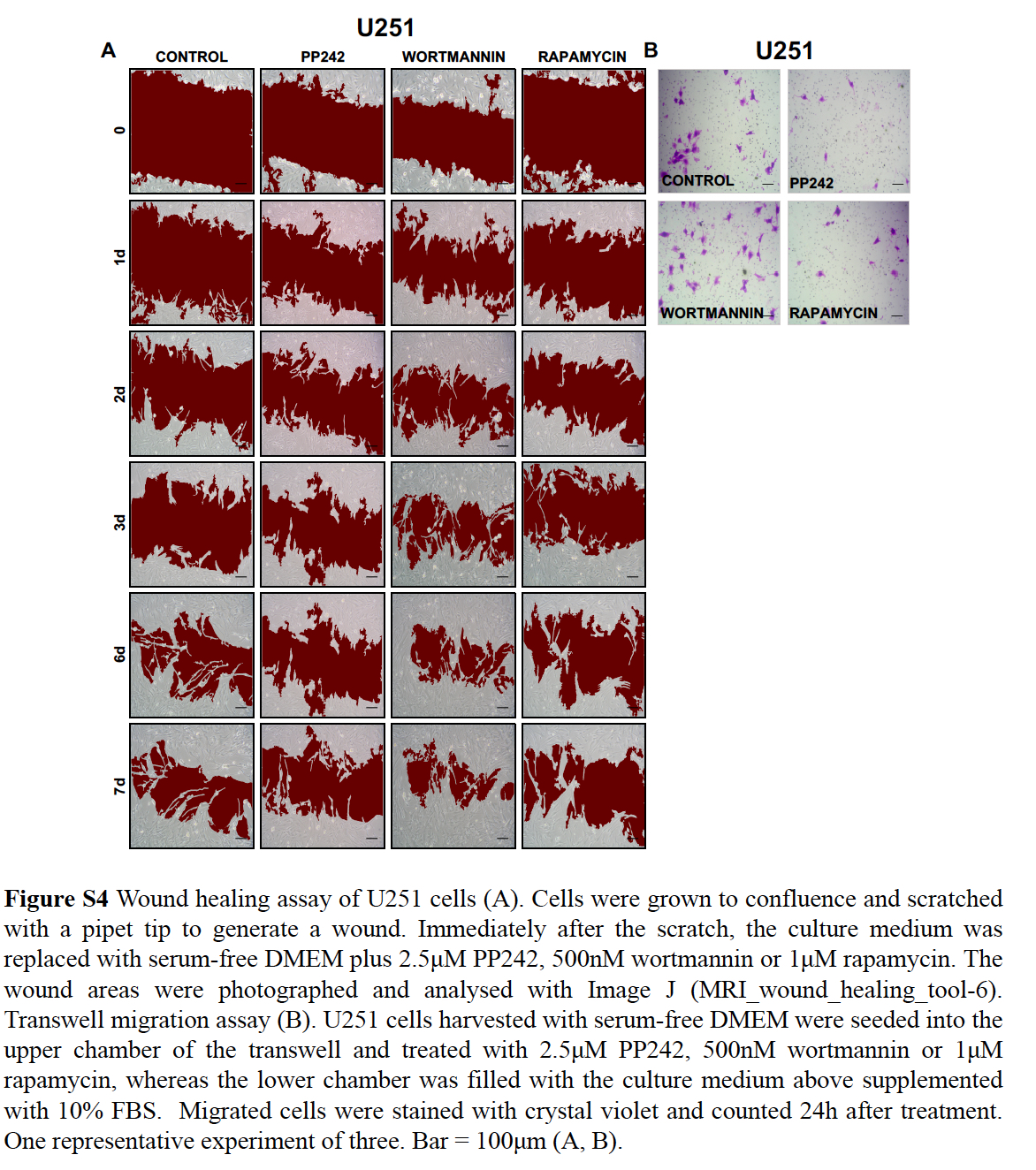

Supplement: Supplementary file 4 [file Image_4.tif]
